# The 7-Year Change in the Prevalence of Insulin Resistance, Inflammatory Biomarkers, and Their Determinants in an Urban South African Population

**DOI:** 10.1155/2020/3781214

**Published:** 2020-05-20

**Authors:** Saarah Fatoma Gadija Davids, Tandi Edith Matsha, Nasheeta Peer, Rajiv Timothy Erasmus, Andre Pascal Kengne

**Affiliations:** ^1^Department of Medicine, University of Cape Town, Cape Town, South Africa; ^2^SAMRC/CPUT Cardiometabolic Health Research Unit, Department of Biomedical Sciences, Cape Peninsula University of Technology, Cape Town, South Africa; ^3^Non-Communicable Diseases Research Unit, South African Medical Research Council, Cape Town, South Africa; ^4^Department of Chemical Pathology, National Health Laboratory Service (NHLS) and Stellenbosch University, Cape Town, South Africa

## Abstract

**Background:**

Insulin resistance (IR) and subclinical inflammation are involved in pathological pathways leading to the development of biological cardiovascular risk factors and subsequent cardiovascular events. Therefore, monitoring these processes can provide advanced information on the trajectory of cardiovascular risk profile of a population and inform prevention and control strategies. We investigated changes in IR and subclinical inflammation in a population from Cape Town, South Africa, between 2008/09 and 2014/16.

**Methods:**

In a total of 2503 (*n* = 797, 2008/09) and (*n* = 1706, 2014/16) participants, IR was calculated using five indices, i.e., insulin fasting, HOMA-IR, QUICKI, McAuley, and Matsuda while subclinical inflammation was measured using usCRP and gamma GT. Linear and logistic regression analyses and interaction tests were conducted.

**Results:**

The mean age of participants was 53.2 (2008/09) and 48.2 (2014/16), respectively. In females, IR prevalence significantly decreased between 2008/09 and 2014/2016 by all indices (*p* ≤ 0.021), while subclinical inflammation prevalence increased from 54.7% (2008/09) to 57.1% (2014/16) based on usCRP and 29.6% to 33.4% based on gamma GT. In a multivariate analysis adjusted for the year of study, age, and gender, prominent factors associated with increased IR or subclinical inflammation were obesity levels measured using waist circumference, glycated haemoglobin, and fasting insulin levels.

**Conclusions:**

Over the 7-year period, subclinical inflammation increased and this was associated with IR and the metabolic syndrome components, both of which are strong predictors of CVDs. The decrease in IR over the year period reflects in part the much younger age in the second survey.

## 1. Introduction

Insulin resistance (IR) and subclinical inflammation are among the pathophysiological derangements involved in the development of cardiometabolic risk factors and related cardiometabolic diseases, the leading cause of death worldwide. Insulin is an anabolic hormone that plays a critical role in the maintenance of glucose homeostasis by promoting glucose transport into muscle and adipose tissue (AT) and inhibiting glucose output by the liver [1]. Resistance to these metabolic actions of insulin (IR) is a major determinant for the development of type 2 diabetes mellitus [2]. In addition, a cluster of other cardiovascular disorders such as dyslipidaemia, obesity, hypertension, and endothelial dysfunction (ED) is associated with IR and is known to interact with each other to promote the development of cardiovascular diseases (CVD) [3]. CVDs are associated with low-grade inflammation; this is demonstrated from increased levels of circulating markers and mediators of inflammation, which in turn are linked to IR [4]. These proinflammatory proteins play a crucial role in the development of IR and subsequent CVDs by activating various inflammatory pathways. For instance, in obesity, macrophages secrete proinflammatory cytokines, tumour necrosis factor alpha (TNF*α*), and interleukin-6 (IL-6) that impairs insulin signalling [5]. TNF*α* reduces the expression of glucose transporter type 4 (GLUT4) which is an insulin-regulated glucose transporter and mainly located in adipocytes, skeletal, and cardiac muscles, resulting in increased circulating triglycerides [6]. IL-6 on the other hand regulates the production of C-reactive protein (CRP), a systemic inflammatory biomarker that has been strongly associated with cardiovascular mortality, hypertension, coronary heart disease, stroke, and diabetes [7].

These pathophysiological derangements involved in the occurrence of CVD develop in apparently healthy individuals in the population for some time, before CVDs and their risk factors occur. Therefore, monitoring pathophysiological biomarkers involved in the development of CVD at a population level can provide early information on the trajectory of CVD burden in the population, even before sizable changes are observed in the population level of CVD risk factors. Such preclinical information can assist in the planning of population-level interventions to timeously curb the rising trajectories. However, there has been a dearth of information on the prevalence and patterns of raised pathophysiological biomarkers linked to the development of CVD in the mixed-ancestry population of Cape Town. Therefore, the aim of this study is to investigate the temporal changes in the prevalence of IR, inflammatory biomarkers, and their determinants in the mixed ancestry population of Bellville South, Cape Town, between 2008/09 and 2014/16.

## 2. Materials and Methods

### 2.1. Study Population and Sampling Procedure

In the mixed ancestry population in Bellville South, Cape Town, two independent cross-sectional surveys were conducted in 2008/09 and 2014/16. Residents who were 18 years and older (2008/09) or 20 years and older (2014/16) were invited to participate in the survey by recruiters who visited each dwelling in the area. Individuals who were bed-ridden or pregnant and underweight and those who were under the age of 20 years in the 2008/2009 survey were excluded. Therefore, the number of participants was 946 in 2008/09 and 1989 in 2014/16. After excluding participants with missing data, who did not fulfil the age criteria, had known diabetes, or were underweight, the final sample size was 797 in 2008/09 and 1706 in 2014/16.

### 2.2. Data Collection

Eligible participants were assessed at a designated research site where trained personnel took informed consent, administered the World Health Organization (WHO) stepwise questionnaire [8], and conducted clinical and biochemical assessments. Anthropometric measurements were performed as per the WHO standardized techniques. Waist and hip circumferences were collected using a nonelastic tape measure while height was measured using a stadiometer to the nearest centimeter [8]. Blood pressure was measured three times in 3-minute intervals [8]. The lowest systolic blood pressure (SBP) and its corresponding diastolic blood pressure (DBP) and pulse readings were used. An oral glucose tolerance test (OGTT) was administered to individuals without known diabetes after an overnight fasting to diagnose type 2 diabetes [9]. Biochemical measurements were sent to an ISO 15189 accredited lab in Cape Town. The following were tested: plasma glucose, plasma insulin, high-density lipoprotein cholesterol (HDL-C), low-density lipoprotein cholesterol (LDL-C), total cholesterol (TC), triglycerides, ultrasensitive C-reactive protein (usCRP), and gamma-glutamyl transferase (gamma GT).

### 2.3. Assessment of Insulin Resistance and Subclinical Inflammation

IR was calculated using five indices: the 75^th^ percentile of fasting insulin, the homeostatic model assessment of IR (HOMA-IR) [10], the 25^th^ percentile QUICKI [11], McAuley index [12], and Matsuda index [13]. The 75^th^ or 25^th^ percentiles were derived from the first survey and the same cut-off points were applied to the two survey populations. Using percentiles specific to each of the sample would lead to differing cut-off points and no change in IR prevalence over time. Subclinical inflammation was assessed using two biomarkers with the following values defining subclinical inflammation: usCRP > 3 mg/L [14] and gamma GT ≥ 38 IU/L [15].

### 2.4. Calculations and Definitions

Body mass index (BMI) was calculated as weight [in kilograms (kg)] divided by height [in meters squared (m^2^)]. For this study, BMI status was divided into three categories, as per the WHO [16]: normal weight (<25.0 kg/m^2^), overweight (25.0 kg/m^2^-29.9 kg/m^2^), and obesity (≥30.0 kg/m^2^). The level of education was divided into two categories: ≤7 years of education (up to completion of primary school) and >7 years of education (secondary schooling and higher). Cotinine level of >10 ng/mL was defined as current smoker. Alcohol consumption was self-reported in the administered questionnaire [8]. Hypertension was defined as SBP ≥ 140 mmHg and/or DBP ≥ 90 mmHg or taking blood pressure lowering medication. The glucose tolerance status was determined using the recommended OGTT test. Prediabetes was defined as fasting plasma glucose between 6.1 and 6.9 mmol/L and/or a 2-hour glucose value between 7.8 mmol/L and 11.1 mmol/L [17]. Screened type 2 diabetes was defined as fasting plasma glucose ≥ 7.0 mmol/L and/or a 2-hour post-OGTT plasma glucose ≥ 11.1 mmol/L. Known type 2 diabetes was self-reported and/or on diabetes medication [17].

### 2.5. Statistical Analysis

Statistica v.13 (TIBCO Software Inc., 2017) and SPSS v.25 (IBM Corp., 2011) were used for the data analyses. Data was tested for normality using normality Q-Q plot. The results are reported as the median (25th and 75th percentiles), mean (standard deviation), and count (percentages). For comparison, the chi-square test, analysis of variance test (ANOVA), or Kruskal-Wallis test was used as appropriate. IR was based on the 75^th^ percentile of fasting insulin (12.5 mIU/L) and HOMA IR (3.1) or the 25^th^ percentile of QUICKI (0.53), McAuley index (6.1), and Matsuda index (4.0). Crude prevalence of IR and inflammatory biomarkers was estimated in all participants as well as in participants only with normoglycemia and who were not taking lipid and/or blood pressure lowering medications. Linear regressions and logistic regression, adjusted for age, gender, and year of study, were used to determine the changes in IR indices, inflammatory biomarkers, and their respective determinants between the two cross-sectional studies. A *p* value < 0.05 was used to characterize statistically significant results.

## 3. Results

### 3.1. Characteristics

The population characteristics by year are presented in Table 1. In both surveys, i.e., 2008/09 and 2014/16, majority of participants were women. The average age in 2014/16 was younger compared to that in 2008/09 (48.2 ± 15.3 years vs. 53.2 ± 14.8 years, *p* < 0.001). Waist circumference (91.4 ± 17.1 cm vs. 96.3 ± 15.0 cm, *p* < 0.001) and hip circumference (103.8 ± 16.5 cm vs. 109.8 ± 14.7 cm, *p* < 0.001) were smaller in 2014/16 vs. 2008/09. The average BMI decreased between 2008/09 and 2014/16 (*p* = 0.013), as did both the prevalence of overweight (27.7% vs. 22.9%) and obesity (44.7% vs. 41.3%). However, blood pressure levels increased significantly between the two periods (Table 1) (both *p* < 0.001). Lipid profiles improved between the two periods: HDL-C increased from 1.2 (1.0, 1.5) mmol/L to 1.3 (1.1; 1.5) mmol/L and LDL-C decreased from 3.6 (3.0; 4.3) to 3.1 (2.5; 3.7) mmol/L (both *p* < 0.001). The abnormal glucose tolerance status significantly decreased between 2008/09 and 2014/16 for prediabetes (23.2% vs. 17.4%) and screen-detected diabetes (17.1% vs. 7.3%) (both *p* < 0.001). Current smokers significantly increased between 2008/09 (43.8%) and 2014/16 (52.9%) (*p* < 0.001), while alcohol use was similar between the periods (*p* = 0.958).

### 3.2. Markers of IR and Inflammation across the Two Time-Points

Markers of subclinical inflammation increased slightly between the periods; i.e., median usCRP increased from 3.6 mg/L in 2008/2009 to 3.9 mg/L in 2014/2016 (*p* = 0.228) and gamma GT from 27 IU/L to 28 IU/L (*p* = 0.011). The indices of IR were generally similar between the two periods (all *p* ≥ 0.05) (Table 1).

### 3.3. Prevalence of Insulin Resistance and Subclinical Inflammation

In the overall study population including screen-detected diabetes, prediabetes, and normoglycemia, IR prevalence significantly decreased between 2008/09 and 2014/2016, fasting insulin from 24.8% in 2008/2009 to 16.9% in 2014/2016, HOMA-IR from 24.8% to15.4%, QUICKI from 23.6% to 13.3%, McAuley from 25.5% to 21.3%, and Matsuda from 25.5% to 18.8% (*p* ≤ 0.021). A similar pattern was observed among women between the periods for all the IR indices (*p* < 0.023). Although the prevalence of IR also decreased in men, it was not significant (*p* ≥ 0.050). In participants with normoglycemia, excluding those taking lipid lowering and/or hypertension medication, the decrease in IR was not significant with McAuley index, from 35.7% in 2008/2009 to 32.8% (*p* = 0.157) in the overall study population. A similar trend was observed in women; however, in men, significant decreases in the prevalence of IR were observed with QUICKI from 27.8% to 19.9% (*p* = 0.034) and Matsuda index from 26.7% to 18.2% (*p* = 0.020) (Table 2).

Subclinical inflammation prevalence increased slightly between 2008/09 and 2014/16, from 54.7% to 57.1% (*p* = 0.264) for usCRP and 29.6% to 33.4% (*p* = 0.055) for gamma GT. Both subclinical inflammatory biomarkers increased in women, but this was significant only for gamma GT (26.2% to 32.1%, *p* = 0.009). In contrast, there were no significant changes in these biomarkers in men between the periods, with both markers (*p* ≥ 0.368), respectively. In the group with normoglycemia without lipid lowering and/or hypertension medication, the prevalence of subclinical inflammation as measured by gamma GT increased significantly in women, with *p* = 0.030 (Table 2).

### 3.4. Determinants of Levels of Markers of Insulin Resistance

From the two surveys, linear regression models for IR and subclinical inflammation, adjusted for year of study, gender, age, and smoking, were determined using log-transformed dependent variables. For IR, the QUICKI, McAuley, and Matsuda indices showed inverse values since their cut-off values are inverse. Using the HOMA-IR index, an increased level of IR was indicated in year of study and age, while men were inversely affected (Table 3). Furthermore, IR markers with the exception of the QUICKI index were positively associated with HbA1c, waist circumference, triglycerides, and gamma GT (all *p* ≤ 0.004), while HDL-C, education level < 7 years, and those who reported current alcohol drinking status were negatively associated, with all *p* ≤ 0.018 (Table 3). Similarly, in a logistic regression adjusted for year of study, age, and gender, the odds of IR in participants in 2014/16 were lower compared to those in 2008/09 (Table 4). Smokers and current drinkers had a lower odds ratio of IR, while usCRP and gamma GT were associated with higher odds of IR, using HOMA-IR (usCRP odds ratio (OR) 2.39, 95% CI 1.88; 3.04 and gamma GT OR: 1.93, 95% 1.54; 2.43) (all *p* ≤ 0.011).

### 3.5. Determinants of Levels of Markers of Subclinical Inflammation

In a secondary analysis presented in Table 5, subclinical inflammation was significantly higher by usCRP in 2014/16 compared with 2008/09 [0.08% (0.04; 0.13), *p* = 0.001]. Additionally, waist circumference, hip circumference, 2-hour glucose, HbA1c, gamma GT, and education < 7 years increased with higher usCRP levels (all *p* ≤ 0.009). Similar findings were observed in the associations with gamma GT with the addition of men gender, current drinkers, systolic blood pressure, fasting glucose, fasting insulin, HDL-C, triglycerides, and usCRP, also associated with subclinical inflammation (*p* ≤ 0.017).

The logistic regression, adjusted for year of study, age, and gender, had shown an almost 1.5-fold higher odds of inflammation in 2014/16 compared to 2008/09 for both markers of inflammation. Insulin resistance, using McAuley and Matsuda indices, had demonstrated an almost 2-fold significantly greater odds of inflammation using usCRP or gamma GT. Additionally, age was significantly associated with inflammation using usCRP, while the lower level of education was associated with inflammation using gamma GT. Subclinical inflammation, using gamma GT, had shown greater odds in smokers (OR 1.26, 95% 1.03; 1.53) and current drinkers (OR 2.47, 95% CI 1.99; 3.06) (Table 6).

## 4. Discussion

Our study found a significant decrease in the overall prevalence of IR over time, which was mainly driven by a significant decrease in women. Using HOMA-IR for example, IR prevalence decreased significantly from 24.8% in 2008/09 to 15.4% in 2014/16 in the overall study population and from 27.7% to 16.6% in women but was not significantly different in men (2008/09: 14.8% to 2014/16: 11.7%). However, using the McAuley index, which is recommended for population-based studies, the decrease in IR prevalence was nonsignificant (35.7% to 32.8%, *p* = 0.157), particularly when only normoglycemic participants excluding those on lipid lowering and/or hypertension medication were analysed separately. Subclinical inflammation marginally increased with both usCRP and gamma GT, indicating increases from 2008/09 to 2014/16. In a multivariable analysis adjusted for the year of study, age, and gender, prominent factors associated with increased IR or subclinical inflammation were abdominal obesity, glycated haemoglobin, and fasting insulin levels.

IR and inflammation are unequivocally associated with the development of type 2 diabetes and CVDs; however, it has also been established that IR even in subjects without type 2 diabetes is connected to high mortality rates, mainly through coronary heart diseases [18]. Estimates from the international diabetes federation have shown that there is a major shift occurring worldwide with regard to type 2 diabetes and CVDs, with predictions of over 100% increases in Africa [19]. In our study, the prevalence of IR ranged from 24.8% to 15.4% using HOMA-IR cut-off of 3.1 between 2008/2009 and 2014/2016. Although our study findings suggest a decrease in IR between the two time periods, these results need to be interpreted with caution. First, the mean age of participants in these two cohorts was significantly different with 2014/2016 overrepresented by younger participants; second, when using the McAuley index, which is recommended for population-based studies, IR nonsignificantly decreased from 35.7% in 2008/2009 to 32.8% in 2014/2016; and lastly, subclinical inflammation increased between the two periods suggesting an increasing risk of CVD.

With regard to comparing the prevalence of IR in our study population with the literature, our findings are somewhat similar to a report from Denmark which reported a prevalence of 17% using a HOMA-IR cut-off of 2.5 [20]. However, our findings contrast with a South African study which reported a prevalence of 55.5% using a cut-off of 2.6 [21]. This varying prevalence could be related to the noncensus on the cut-off criteria for the definition of IR. According to the WHO [22], the ≥75^th^ percentile value is recommended for the definition of IR; however, various cut-offs are reported in the literature.

usCRP is a well-established inflammatory biomarker for predicting future risk of CVD events [22, 23]. Thus, the measurement of usCRP has been used in the identification of high-risk individuals who may benefit from therapeutic interventions [23]. Another biomarker that has attracted interest is gamma GT activity which is widely accepted for the diagnosis of liver and obstructive biliary diseases as well as excessive alcohol consumption. Evidence from epidemiological studies has shown that gamma GT activity is associated with type 2 diabetes, metabolic syndrome, atherosclerosis, and CVDs [24]. In a meta-analysis involving 67 905 individuals, gamma GT activity was significantly associated with the metabolic syndrome in individuals in the highest versus lowest thirds of baseline gamma GT activity [25]. Indeed, in our study, dyslipidaemia parameters (triglycerides, LDL-C, and HDL-C), glucose homeostasis (HbA1c, fasting glucose, and insulin), SBP, age, and gender (men) were associated with increased gamma GT levels. Similarly, other epidemiological studies have reported these findings [26, 27]. For example, a study of 1680 Han Chinese patients found that gamma GT was positively correlated with waist circumference, fasting plasma glucose, triglycerides, blood pressure levels, and the metabolic syndrome [27]. Several mechanisms underlying the association between gamma GT activity and the metabolic syndrome or its components have been suggested. One example is the association between gamma GT activity and IR whereby IR is viewed as a bridging mechanism linking gamma GT with CVD and coronary heart diseases [28]. Indeed, in our study, we have demonstrated that both usCRP and gamma GT had an almost 2.5-fold higher odds for IR.

The strength of this study is that the two surveys were conducted in the population from the same geographical area, using similar procedures. However, the study has the following limitations: (i) it consisted of only two cross-sectional surveys, which prevents reliable assessment of time trends; (ii) the second study was not a follow-up but rather another cross-sectional study assessing new participants from the area; (iii) there was a low proportion of men in both studies (24%), a common problem in SA research [29]; (iv) cotinine levels were used to distinguish smokers from nonsmokers; thus, past smokers and those using nicotine gum could not be identified objectively, which is important as previous studies reported that past smokers and those using nicotine gum had increased levels of IR and inflammatory biomarkers [30]; (v) although numerous IR surrogates were used, only the McAuley index is recommended for population-based studies.

## 5. Conclusion

Our findings have shown increases in subclinical inflammation over a 7-year period, and this was associated with IR and the metabolic syndrome components, both of which are strong predictors of CVDs. Given the estimated increases in the incidence of T2DM and CVD in South Africa and Africa in general, monitoring of these biomarkers involved in the pathophysiological development of CVD is important. It can provide early information on the trajectory of the CVD burden in the population, even before substantial changes are observed in population levels of CVD risk factors.

## Figures and Tables

**Table 1:** Characteristics of participants in 2008/09 and 2014/16.

Characteristics	2008/09 (*n* = 797)	2014/16 (*n* = 1706)	*p* value
	(*n* = 797) Median (25^th^; 75^th^ percentiles)	(*n* = 1706) Median (25^th^; 75^th^ percentiles)	
*Gender*			0.114
Women, *n* (%)	620 (77.8)	1278 (74.9)	
Men, *n* (%)	177 (22.2)	428 (25.1)	
Age, year^∗^	53.2 ± 14.8	48.2 ± 15.3	<0.001
*Level of education*			
Education level ≤ 7 years, *n* (%)	271 (34.2)	559 (33.0)	0.553
Education level > 7 years, *n* (%)	522 (65.8)	1136 (67)	0.553
*Alcohol use*			
Current drinker, *n* (%)	563 (71.4)	1213 (71.5)	0.958
Nondrinker, *n* (%)	225 (28.6)	483 (28.5)	0.958
Cotinine (ng/mL)	10 (9; 311)	48.2 (10; 268)	<0.001
*Tobacco use*			
Nonsmoker, *n* (%)	447 (56.2)	779 (47.1)	<0.001
Smoker, *n* (%)	348 (43.8)	874 (52.9)	<0.001
Anthropometry			
Body mass index (kg/m^2^)^∗^	29.64 ± 7.24	28.83 ± 8.1	0.013
*BMI status*			
Normal (18.5 kg/m^2^ to 25 kg/m^2^), *n* (%)	218 (27.7)	603 (35.8)	<0.001
Overweight (25 kg/m^2^ to 29.9 kg/m^2^), *n* (%)	218 (27.7)	385 (22.9)	0.009
Obese (≥30 kg/m^2^), *n* (%)	352 (44.7)	696 (41.3)	0.109
Waist circumference (cm)^∗^	96.3 ± 15.0	91.4 ± 16.9	<0.001
Hip circumference (cm)^∗^	109.8 ± 14.7	103.83 ± 16.5	<0.001
Waist-to-hip ratio^∗^	0.9 ± 0.1	0.88 ± 0.09	0.504
Systolic blood pressure (mmHg)^∗^	123 ± 19	126 ± 24	<0.001
Diastolic blood pressure (mmHg)^∗^	75 ± 12	82 ± 14	<0.001
Fasting glucose (mmol/L)	5.6 (5.0;6.5)	5.0 (4.6;5.6)	<0.001
2 hr glucose (mmol/L)	6.8 (5.7;8.7)	6.1 (4.9; 7.6)	<0.001
HBA1c (mmol/mol)^∗^	65 ± 10	63 ± 9	<0.001
HBA1c (%)^∗^	5.9 ± 0.9	5.8 ± 0.8	<0.001
Fasting insulin (mIU/L)	6.6 (2.7; 12.5)	6.6 (4.2; 10.4)	0.414
2 hr insulin (mIU/L)	38.1 (19.5; 72.2)	38.8 (20.7; 71.9)	0.829
Normoglycemia, *n* (%)	474 (59.7)	1278 (75.3)	<0.001
Pre-diabetes, *n* (%)	184 (23.2)	296 (17.4)	<0.001
Screened-detected diabetes, *n* (%)	136 (17.1)	124 (7.3)	<0.001
HDL-C (mmol/L)	1.2 (1.0; 1.5)	1.3 (1.1; 1.5)	<0.001
LDL-C (mmol/L)	3.6 (3.0; 4.3)	3.1 (2.5; 3.7)	<0.001
Triglycerides (mmol/L)	1.2 (0.9; 1.7)	1.5 (0.8; 1.7)	0.620
Ultra-sensitive C-reactive protein (mg/L)	3.6 (0.9; 9.4)	3.9 (1.5; 8.6)	0.228
Gamma-glutamyl transferase (IU/L)	27 (19; 42)	28 (20; 45)	0.011
HOMA-IR	1.6 (0.6; 3.1)	1.4 (0.9; 2.4)	0.859
QUICKI	0.6 (0.5; 0.9)	0.7 (0.6; 0.8)	0.124
McAuley index	7.9 (6.1; 10.7)	7.9 (6.4; 9.4)	0.050
Matsuda index	7.9 (4.0; 17.8)	8.4 (4.8; 15.3)	0.685

^∗^Mean ± SD; screen-detected diabetes: newly diagnosed diabetics; HDL-C: high-density lipoprotein cholesterol; LDL-C: low-density lipoprotein cholesterol; HOMA-IR: homeostatic model assessment of insulin resistance.

**Table 2:** Prevalence of IR and inflammation in all participants and in those with normoglycemia and not on lipid lowering and/or hypertension medication.

	Overall			Women			Men			Gender ∗ year of study *p* interaction
	2008/09 *n* (%)	2014/16 *n* (%)	*p* value	2008/09 *n* (%)	2014/16 *n* (%)	*p* value	2008/09 *n* (%)	2014/16 *n* (%)	*p* value	
Insulin resistance: all participants										
Fasting insulin	196 (24.8)	278 (16.9)	<0.001	174 (28.4)	231 (18.7)	<0.001	22 (12.5)	47 (11.4)	0.706	0.939
HOMA-IR	196 (24.8)	253 (15.4)	<0.001	170 (27.7)	205 (16.6)	<0.001	26 (14.8)	48 (11.7)	0.296	0.375
QUICKI	186 (23.6)	218 (13.3)	<0.001	158 (25.8)	176 (14.3)	<0.001	28 (15.9)	42 (10.2)	0.050	0.550
McAuley index	201 (25.5)	348 (21.3)	0.021	167 (27.2)	275 (22.4)	0.023	34 (19.5)	73 (18.0)	0.667	0.369
Matsuda index	200 (25.5)	299 (18.8)	<0.001	174 (28.6)	255 (21.5)	0.001	26 (14.8)	44 (10.9)	0.194	0.119
Insulin resistance: normoglycemia and not on lipid lowering and/or hypertension medication										
Fasting insulin	247 (31.3)	413 (25.1)	0.001	211 (34.4)	343 (27.8)	0.004	36 (20.5)	70 (17.0)	0.317	0.765
HOMA-IR	288 (36.5)	451 (27.4)	<0.001	248 (40.5)	377 (30.6)	<0.001	40 (22.7)	74 (18.0)	0.181	0.455
QUICKI	323 (40.9)	504 (30.6)	<0.001	274 (44.7)	422 (34.2)	<0.001	49 (27.8)	82 (19.9)	0.034	0.548
McAuley index	281 (35.7)	535 (32.8)	0.157	233 (38.0)	430 (35.1)	0.216	48 (27.6)	105 (25.9)	0.678	0.236
Matsuda index	310 (39.5)	542 (34.1)	0.009	263 (43.3)	469 (39.5)	0.123	47 (26.7)	73 (18.2)	0.020	0.170
Sub-clinical inflammation: all participants										
usCRP	436 (54.7)	967 (57.1)	0.264	351 (56.6)	772 (60.8)	0.079	85 (48.02)	195 (45.9)	0.631	0.092
Gamma GT	235 (29.6)	567 (33.4)	0.055	162 (26.2)	408 (32.1)	0.009	73 (42.2)	159 (37.3)	0.368	0.158
Sub-clinical inflammation: normoglycemia and not on lipid lowering and/or hypertension medication										
usCRP	160 (46.2)	480 (49.5)	0.3	124 (47.9)	356 (53.1)	0.157	36 (41.4)	124 (41.5)	0.625	0.217
Gamma GT	83 (24.1)	276 (28.4)	0.12	50 (19.4)	176 (26.2)	0.03	33 (37.9)	100 (33.3)	0.372	0.166

^∗^Interaction between men and women; HOMA-IR: homeostatic model assessment of insulin resistance; usCRP: ultrasensitive C-reactive protein; gamma GT: gamma-glutamyl transferase.

**Table 3:** Year of study-, gender-, age-, and smoking-adjusted linear regression models for determinants of insulin resistance in all participants.

	Model 1: log insulin fasting^∗^				Model 2: log HOMA-IR^∗^				Model 3: log QUICKI index^∗^				Model 4: log McAuley index^∗^				Model 5: log Matsuda index^∗^			
	B	95% confidence interval		*p* value	B	95% confidence interval		*p* value	B	95% confidence interval		*p* value	B	95% confidence interval		*p* value	B	95% confidence interval		*p* value
		Lower	Upper			Lower	Upper			Lower	Upper			Lower	Upper			Lower	Upper	
Year of study (2014/16)	0.16	0.13	0.20	<0.001	0.06	0.03	0.10	<0.001	-0.39	-0.51	-0.26	<0.001	-0.05	-0.06	-0.04	<0.001	-0.09	-0.12	-0.06	<0.001
Gender (men)	-0.15	-0.19	-0.11	<0.001	-0.15	-0.18	-0.11	<0.001	0.18	0.04	0.32	0.012	0.04	0.03	0.05	<0.001	0.21	0.18	0.25	<0.001
Age	0.43	0.30	0.55	<0.001	0.40	0.28	0.51	<0.001	-0.39	-0.85	-0.05	0.085	-0.12	-0.16	-0.08	<0.001	-0.21	-0.33	-0.10	<0.001
Current smokers	-0.08	-0.12	-0.05	<0.001	-0.08	-0.11	-0.05	<0.001	0.09	-0.02	0.21	0.122	0.02	0.01	0.03	<0.001	0.12	0.09	0.15	<0.001
Education (<7 years)	-0.05	-0.09	-0.02	0.002	-0.04	-0.08	-0.01	0.007	0.01	-0.11	0.13	0.829	0.01	0.01	0.02	0.002	0.04	0.01	0.07	0.012
Current drinkers	-0.06	-0.10	-0.02	0.003	-0.05	-0.08	-0.01	0.007	0.03	-0.11	0.16	0.707	0.02	0.01	0.03	0.003	0.05	0.01	0.08	0.006
Waist circumference	0.85	0.65	1.04	<0.001	0.87	0.68	1.05	<0.001	-0.82	-1.50	-0.15	0.017	-0.24	-0.29	-0.18	<0.001	-0.87	-1.05	-0.70	<0.001
Hip circumference	-0.15	-0.34	0.05	0.142	-0.15	-0.33	0.03	0.107	0.58	-0.10	1.27	0.094	0.04	-0.01	0.09	0.142	0.22	0.05	0.40	0.014
Systolic blood pressure	0.01	-0.07	0.09	0.792	0.03	-0.04	0.10	0.369	0.00	-0.26	0.27	0.993	0.00	-0.02	0.02	0.792	-0.05	-0.12	0.02	0.142
HbA1c	2.95	0.92	5.02	0.004	8.67	6.66	10.72	<0.001	-9.78	-15.87	-3.25	0.004	-0.81	-1.36	-0.26	0.004	-8.19	-9.92	-6.41	<0.001
HDL-C^∗^	-0.28	-0.51	-0.05	0.018	-0.29	-0.50	-0.07	0.010	0.06	-0.75	0.87	0.877	0.08	0.01	0.14	0.018	0.35	0.13	0.56	0.001
LDL-C^∗^	0.29	-0.10	0.68	0.150	0.26	-0.11	0.62	0.172	-0.46	-1.84	0.91	0.509	-0.08	-0.19	0.03	0.150	-0.27	-0.62	0.09	0.148
Triglycerides^∗^	0.31	0.19	0.43	<0.001	0.31	0.20	0.42	<0.001	-0.20	-0.62	0.22	0.342	-0.40	-0.43	-0.36	<0.001	-0.34	-0.45	-0.23	<0.001
usCRP^∗^	-0.02	-0.05	0.02	0.323	-0.01	-0.04	0.02	0.505	0.07	-0.03	0.18	0.177	0.00	0.00	0.01	0.323	0.00	-0.03	0.03	0.945
Gamma GT^∗^	0.10	0.04	0.15	<0.001	0.13	0.07	0.18	<0.001	-0.16	-0.36	0.03	0.098	-0.03	-0.04	-0.01	<0.001	-0.13	-0.19	-0.08	<0.001

^∗^Log transformed; HOMA-IR: homeostatic model assessment of insulin resistance; HDL-C: high-density lipoprotein cholesterol; LDL-C: low-density lipoprotein cholesterol; usCRP: ultrasensitive C-reactive protein; gamma GT: gamma-glutamyl transferase.

**Table 4:** Age-, sex-, and year of study-adjusted odds ratios (with 95% confidence intervals) for the determinants of insulin resistance in all participants.

	Model 1: insulin fasting				Model 2: HOMA-IR				Model 3: QUICKI index				Model 4: McAuley index				Model 5: Matsuda index			
	Odds ratio	95% confidence interval		*p* value	Odds ratio	95% confidence interval		*p* value	Odds ratio	95% confidence interval		*p* value	Odds ratio	95% confidence interval		*p* value	Odds ratio	95% confidence interval		*p* value
		Lower	Upper			Lower	Upper			Lower	Upper			Lower	Upper			Lower	Upper	
*Year of study*																				
2008/09	1				1				1				1				1			
2014/16	0.57	0.46	0.71	<0.001	0.51	0.41	0.64	<0.001	0.45	0.36	0.57	<0.001	0.78	0.63	0.97	0.025	0.67	0.54	0.84	<0.001
*Gender*																				
Women	1				1				1				1				1			
Men	0.58	0.43	0.77	<0.001	0.67	0.50	0.89	0.006	0.72	0.54	0.97	0.031	0.83	0.64	1.06	0.137	0.53	0.4	0.71	<0.001
*Age*	0.99	0.98	1.00	0.002	0.99	0.99	1.00	0.106	0.99	0.98	1.00	0.031	1.00	1.00	1.01	0.538	1.00	0.99	1.01	0.548
*Tobacco use*																				
Nonsmoker	1				1				1				1				1			
Smoker	0.66	0.52	0.83	<0.001	0.64	0.50	0.80	<0.001	0.72	0.57	0.92	0.009	0.69	0.56	0.86	0.001	0.50	0.39	0.63	<0.001
*Level of education*																				
<7 years	1				1				1				1				1			
≥7 years	1.26	0.99	1.61	0.063	1.14	0.89	1.45	0.310	1.13	0.88	1.46	0.349	1.09	0.87	1.37	0.461	1.21	0.95	1.54	0.115
*Alcohol use*																				
Nondrinker	1				1				1				1				1			
Current drinker	0.66	0.5	0.88	0.004	0.7	0.52	0.92	0.011	0.66	0.49	0.88	0.005	0.74	0.57	0.95	0.02	0.62	0.47	0.83	0.001
*BMI status*																				
Normal	1				1				1				1				1			
Overweight	1.18	0.88	1.56	0.518	1.07	0.80	1.44	0.778	1.19	0.88	1.60	0.538	1.08	0.82	1.42	0.501	1.15	0.86	1.54	0.563
Obese	1.11	0.86	1.43	0.518	1.1	0.85	1.41	0.778	1.08	0.83	1.41	0.538	1.15	0.91	1.46	0.501	1.12	0.87	1.44	0.563
*usCRP*																				
No	1				1				1				1				1			
Yes	2.28	1.81	2.88	<0.001	2.39	1.88	3.04	<0.001	2.16	1.69	2.77	<0.001	2.05	1.65	2.55	<0.001	2.38	1.89	3.01	<0.001
*Gamma GT*																				
No	1				1				1				1				1			
Yes	1.75	1.4	2.19	<0.001	1.93	1.54	2.43	<0.001	2.08	1.65	2.64	<0.001	2.33	1.89	2.88	<0.001	2.23	1.78	2.79	<0.001

HOMA-IR: homeostatic model assessment of insulin resistance; usCRP: ultrasensitive C-reactive protein; gamma GT: gamma-glutamyl transferase.

**Table 5:** Year of study-, gender-, age-, and smoking-adjusted linear regression models for determinants of inflammation in all participants.

	Model 1: log usCRP				Model 2: log gamma GT			
	*B*	95% confidence interval		*p* value	*B*	95% confidence interval		*p* value
		Lower	Upper			Lower	Upper	
Year of study (2014/16)	0.08	0.04	0.13	0.001	0.03	0.00	0.06	0.033
Gender (men)	-0.04	-0.09	0.02	0.200	0.08	0.05	0.11	<0.001
Age	0.09	-0.08	0.26	0.316	-0.28	-0.37	-0.18	<0.001
Current smokers	0.10	0.05	0.14	<0.001	0.06	0.03	0.08	<0.001
Education (<7 years)	0.07	0.03	0.12	0.002	0.03	0.00	0.05	0.040
Current drinkers	-0.05	-0.10	0.00	0.040	0.11	0.08	0.14	<0.001
Waist circumference	0.75	0.49	1.01	<0.001	0.33	0.19	0.48	<0.001
Hip circumference	0.43	0.17	0.68	0.001	-0.29	-0.43	-0.14	<0.001
Systolic blood pressure	-0.04	-0.14	0.06	0.414	0.11	0.05	0.17	<0.001
Fasting glucose^∗^	-0.36	-0.71	-0.01	0.043	0.24	0.04	0.43	0.017
2 hr glucose^∗^	0.41	0.21	0.60	<0.001	0.30	0.19	0.40	<0.001
HbA1c	4.88	1.16	8.74	0.010	-1.97	-3.92	0.02	0.052
Fasting insulin^∗^	-0.01	-0.07	0.06	0.872	0.05	0.02	0.09	0.004
2 hr insulin^∗^	-0.03	-0.09	0.04	0.442	-0.01	-0.04	0.03	0.673
HDL-C^∗^	-0.65	-0.86	-0.44	<0.001	0.59	0.47	0.70	<0.001
LDL-C^∗^	-0.09	-0.25	0.08	0.296	-0.10	-0.19	-0.01	0.031
Triglycerides^∗^	-0.13	-0.25	-0.02	0.022	0.30	0.24	0.36	<0.001
C-reactive protein^∗^	—	—	—	—	0.08	0.06	0.10	<0.001
Gamma GT^∗^	0.26	0.19	0.33	<0.001	—	—	—	—

^∗^Log transformed; HDL-C: high-density lipoprotein cholesterol; LDL-C: low-density lipoprotein cholesterol; usCRP: ultrasensitive C-reactive protein; gamma GT: gamma-glutamyl transferase.

**Table 6:** Age-, sex-, and year of study-adjusted ORs (with 95% CIs) for the determinants of inflammation in all participants.

	Model 1: usCRP				Model 2: gamma GT			
	Odds ratio	95% confidence interval		*p* value	Odds ratio	95% confidence interval		*p* value
		Lower	Upper			Lower	Upper	
*Year of study*								
2008/09	1				1			
2014/16	1.23	1.02	1.49	0.029	1.32	1.07	1.61	0.008
*Gender*								
Female	1				1			
Male	0.64	0.52	0.78	<0.001	1.30	1.05	1.61	0.016
*Age*	1.01	1.00	1.01	0.021	1.00	1.00	1.01	0.239
*Tobacco use*								
Nonsmoker	1				1			
Smoker	1.03	0.86	1.24	0.722	1.26	1.03	1.53	0.024
*Level of education*								
<7 years	1				1			
≥7 years	0.83	0.68	1.01	0.056	0.76	0.62	0.94	0.011
*Alcohol use*								
Nondrinker	1				1			
Current drinker	0.82	0.67	1.01	0.062	2.47	1.99	3.06	<0.001
*BMI status*								
Normal	1				1			
Overweight	0.86	0.68	1.07	0.064	0.87	0.68	1.11	0.315
Obese	1.11	0.91	1.35	0.064	0.86	0.69	1.06	0.315
*Insulin fasting*								
No	1				1			
Yes	1.19	0.76	1.86	0.452	0.63	0.39	1.01	0.055
*HOMA-IR*								
No	1				1			
Yes	1.53	0.79	2.99	0.210	0.82	0.43	1.57	0.551
*QUICKI index*								
No	1				1			
Yes	0.71	0.40	1.28	0.258	1.58	0.90	2.74	0.101
*McAuley index*								
No	1				1			
Yes	1.40	1.05	1.87	0.022	2.12	1.59	2.84	<0.001
*Matsuda index*								
No	1				1			
Yes	1.76	1.25	2.47	0.001	1.78	1.27	2.50	0.001

HOMA-IR: homeostatic model assessment of insulin resistance; usCRP: ultrasensitive C-reactive protein; gamma GT: gamma-glutamyl transferase.

## Data Availability

The datasets generated and/or analysed during the current study are available from the principal investigator (TEM) on reasonable request.
